# *PTPN22* Gene Polymorphisms Are Associated with Susceptibility to Large Artery Atherosclerotic Stroke and Microembolic Signals

**DOI:** 10.1155/2019/2193835

**Published:** 2019-05-05

**Authors:** Lingyan Zhou, Kun Wang, Junhong Wang, Zhenfeng Zhou, Yufei Cheng, Xudong Pan, Aijun Ma

**Affiliations:** Department of Neurology, The Affiliated Hospital of Qingdao University, Qingdao 266000, China

## Abstract

Large artery atherosclerotic stroke (LAAS) is the most common ischemic stroke (IS) subtype, and microemboli may be clinically important for indicating increased risk of IS. The inflammatory process of atherosclerosis is well known, and lymphoid phosphatase (Lyp), which is encoded by the protein tyrosine phosphatase nonreceptor type 22 (*PTPN22*) gene, plays an important role in the inflammatory response. Our study was intended to evaluate the relationship between *PTPN22* gene and LAAS and microembolic signals (MES). Three loci of the *PTPN22* gene (rs2476599, rs1217414, and rs2488457) were analyzed in 364 LAAS patients and 369 control subjects. A genotyping determination was performed using the TaqMan assay. The G allele of rs2488457 might be related to a higher risk for developing LAAS and MES (odds ratio (OR) = 1.456, 95% confidence interval (CI) 1.156-1.833, *P* = 0.001; OR = 1.652, 95% CI 1.177-2.319, *P* = 0.004, respectively). In the LAAS group, the prevalence of the GTG haplotype was higher (*P* < 0.001) and the prevalence of the GCC haplotype was lower (*P* = 0.001). An interaction analysis of rs2488457 with smoking showed that smokers with the CG/GG genotypes had a higher risk of LAAS, compared to nonsmokers with the rs2488457 CC genotype (OR = 2.492, 95% CI 1.510–4.114, *P* < 0.001). Our research indicated that the *PTPN22* rs2488457 might be related to the occurrence of LAAS and MES in the Han Chinese population. In addition, the rs2488457 polymorphism and the environmental factor of smoking jointly influenced the susceptibility of LAAS.

## 1. Introduction

Stroke is considered the most general cause of adult-acquired deformity [1]. In all stroke events worldwide, ischemic stroke (IS) comprises 80−85% [1, 2]. It is logical that ischemic stroke is a complicated and multifactorial disease that is influenced by several risk factors, including environmental and genetic factors [3]. In the age of precision medicine, much research focused on the relationship between genetic factors, such as single-nucleotide polymorphisms (SNPs), and susceptibility to IS [4]. On the basis of the TOAST (Trial of ORG 10172 in Acute Stroke Treatment) [5] classification system, the main subtype of ischemic stroke is large artery atherosclerotic stroke (LAAS) [6]. Therefore, it will be of great significance to study the relationship between SNPs and LAAS for the prevention, diagnosis, treatment, and prognosis of IS [7].

It is generally accepted that LAAS is mainly triggered by atherosclerosis. The inflammatory process of atherosclerosis has been recognized as early as the 19th century. Substantial evidence has suggested that the inflammatory mechanisms involved in the pathogenesis of atherosclerosis are regulated by both adaptive and innate immunity [8–10]. The protein tyrosine phosphatase nonreceptor (*PTPN22*) gene is located on chromosome 1p13.2 and encodes the cytoplasmic lymphoid phosphatase (Lyp). Lyp is required for the immune system function to be well balanced [11, 12]. In recent years, the *PTPN22* gene and its susceptibility to a variety of inflammatory diseases have been recognized [13–16]. Furthermore, genetic variants of *PTPN22* increased the risk of atherosclerosis in people with autoinflammatory disorders [17, 18]. In patients with large arterial occlusive disease, microemboli that are released from atherosclerotic plaques can result in acute ischemic stroke [19]. Microembolic signals (MES) can be detected through the transcranial Doppler ultrasonography (TCD). MES suggest that atherosclerotic plaques may be unstable and may be potential risk markers for ischemic stroke [20]. In addition, smoking and drinking are considered significant environmental risk factors for LAAS. Therefore, our research was designed to evaluate whether three SNPs of the *PTPN22* gene are correlated with LAAS and MES as well as the interaction of these SNPs with smoking and drinking.

## 2. Materials and Methods

### 2.1. Study Population

We recruited 364 LAAS patients and 369 healthy control subjects from unrelated ethnic Han Chinese populations. LAAS patients were diagnosed by two experienced neurologists on the basis of TOAST [5] criteria and had lesions that were restricted to the middle cerebral artery or internal carotid artery areas. All the patients were hospitalized in the Neurology Department of the Affiliated Hospital of Qingdao University between 2013 and 2017. The inclusion criteria of the cases were the presence of ischemic stroke, which was confirmed by magnetic resonance imaging (MRI) or computer tomography (CT), and the presence of cardiac and cerebral vascular lesions, which were confirmed by echocardiography, TCD, brain magnetic resonance angiography (MRA), or whole-brain digital subtraction angiography (DSA). All patients were subjected to microembolism monitoring. The patients were divided into MES-negative and MES-positive groups according to the presence of MES. The exclusion criteria were patients who were diagnosed with any other subtype of IS (small artery occlusion, cardioembolic stroke, stroke of undetermined etiology, and stroke of other determined etiology) or who had severe infection, severe heart disease (recent myocardial infarction, angina pectoris disease, and valvular heart disease), severe hepatic and renal insufficiency, chronic inflammation, a malignant tumor, or autoimmune disease. Healthy control subjects were enrolled from the health examination center at our hospital during the same time period. The inclusion criteria were as follows: participants who had no clinical features or neuroimaging evidence of cerebral infarction, nor obvious cerebral atherosclerosis or angiostenosis, nor any history of ischemic stroke. The exclusion criteria were similar to the criteria applied to the LAAS group.

The research was carried out according to the ethical guidelines within the Helsinki declaration for experiments involving human participants. This study protocol was authorized by the ethical committee of the Affiliated Hospital of Qingdao University (QDDXYXYFSXY-2014-005). Informed consent was acquired from each participant in this study.

### 2.2. Data Collection

Clinical data and demographic information from all the subjects were collected by well-trained investigators. Structured questionnaire surveys were administered to acquire the general statuses of the subjects, including body mass index (BMI); age; sex; history of smoking, drinking, hypertension, diabetes, coronary artery disease (CAD), and dyslipidemia; and family history of ischemic stroke events. After one night of fasting, antecubital venous blood (4 mL) samples were collected from all participants and were later placed into EDTA tubes followed by a 10-minute centrifugation at 3000 rpm. Levels of serum blood glucose (GLU), triglycerides (TG), total cholesterol (TC), low-density lipoproteins (LDL), and high-density lipoproteins (HDL) were measured in the clinical laboratory of our hospital.

### 2.3. SNP Selection

*PTPN22* gene was selected based on exploring previously reported genes that might be involved in inflammatory responses or related to inflammatory diseases. To the best of our knowledge, the association between *PTPN22* gene and LAAS and MES has not been reported so far. According to minimum allele frequency, minor allele frequency (MAF) was greater than 5% in the Chinese Beijing Han population (CHB) from the 1000 Genomes Browser and previously reported gene loci that might be related to inflammatory responses. Finally, three SNPs (rs2476599, rs1217414, and rs2488457) were selected.

### 2.4. Genotyping

Following the manufacturer's instructions of TIANamp Blood DNA Kit (Tiangen Biotech, Beijing, China), we extracted genomic DNA from the blood samples. TaqMan technology was used to explore the *PTPN22* rs2476599, rs1217414, and rs2488457 gene polymorphisms. The specific primers and probes were designed and synthesized by the Sangon Biotech Company (Shanghai, China). The sequences of the primers and probes are shown in Supplementary 1. Each PCR reaction consisted of 5 *μ*L 2x TaqMan Fast qPCR Master Mix (Sangon Biotech, Shanghai, China), 0.25 *μ*L forward and 0.25 *μ*L reverse primer (10 *μ*M), 0.2 *μ*L (10 *μ*M) FAM-labeled probe, 0.2 *μ*L (10 *μ*M) VIC-labeled probe, 2.0 *μ*L template DNA, and 2.1 *μ*L double-distilled H_2_O (ddH_2_O). The PCR cycling conditions were as follows: predegeneration for 3 min at 94°C and then denaturation at 94°C for 5 s and 40 cycles of annealing and extension for 30 s at 60°C. Reactions were performed on the LightCycler® 480 II Real-Time PCR System (Roche Diagnostics, Manheim, Germany) according to the manufacturer's protocols. Afterwards, we analyzed the genotypes on the LightCycler® 480 software version 1.5.0. To ensure accuracy of genotyping, 20% of the samples for each polymorphism were randomly selected for direct sequencing.  Supplementary 2 shows the sequences of primers for DNA sequencing. The results that were achieved by the two methods were absolutely concordant.

### 2.5. Microembolic Signal Monitoring

Within 72 hours of the onset of stroke, TCD (EMS-9EB × 2P Doppler box/EMS-9EB multidrop × 4.2 MHz probe) was used for monitoring the MES in all patients. The 2 MHz probe was fixed to the patient's head, and the MES monitoring was performed in the initial segment and distal segment of the symptomatic middle cerebral artery (MCA) (distance between two points, ≥6 mm; sampling depth, 50–65 mm; sample volume, 8 mm; MES threshold, ≥5 dB; and monitoring time, 60 min). The criteria for microembolic signals were based on a study by Ringelstein et al. [21]. Microembolism monitoring was conducted by two trained and experienced neurologists.

### 2.6. Statistical Analysis

Count data are presented as the number of cases and percentages and were analyzed by using chi-squared test. Quantitative variables were recorded as the means ± standard deviations (SDs) with normal distributions, and Student's *t*-test was applied to analyze the differences. Hardy-Weinberg equilibrium (HWE) testing was carried out by using chi-squared tests for each locus. Pearson's *χ* ^2^ test or Fisher's exact test was used for comparisons of genotypes and allele frequencies. Logistic regression analyses were performed to compute odds ratios (OR) with 95% confidence intervals (CI) after adjusting the covariates (sex, age, history of smoking and drinking, diabetes, hypertension, dyslipidemia, and family history of ischemic stroke events) to assess the correlation between LAAS and *PTPN22* gene polymorphisms. Linkage disequilibrium (LD) and haplotype distributions were estimated using the SHEsis software platform [22, 23]. Tests for interactions with drinking or smoking were only focused on the SNPs that were significantly associated with LAAS. All statistical analyses were performed using the SPSS 20.0 software. *P* value < 0.05 (two-tailed) was considered statistically significant. Power calculation was performed at the 0.05 level of type I error probability using the G^∗^Power software version 3.1.

## 3. Results

### 3.1. Characteristics of the Subjects

Table 1 summarizes the clinical characteristics of LAAS patients and control subjects. There were no differences between two cohorts in the aspects of age, sex, BMI, history of CAD, and the levels of LDL, TG, and HDL (*P* > 0.05). The levels of GLU and TC, diabetes, hypertension, dyslipidemia, alcohol consumption, family history of ischemic stroke events, and smoking are higher in the LAAS group (*P* < 0.05).

### 3.2. Association between PTPN22 Gene Polymorphisms and LAAS

The distributions of the alleles and genotypes were in accordance with Hardy-Weinberg equilibrium in both groups (*P* > 0.05). When compared to the reference (CC), we observed that the GG genotype of rs2488457 was associated with a significantly increased risk of LAAS after controlling for other covariates selected in our study (adjusted OR = 2.275, 95% CI 1.395-3.709, *P* = 0.001). Furthermore, patients carrying the G allele showed an increased risk of LAAS compared with carriers of the C allele (adjusted OR = 1.456, 95% CI 1.156-1.833, *P* = 0.001). However, the genotypes and allele frequencies for rs1217414 and rs2476599 were not significantly different (Table 2). The statistical power for detecting the relation between rs2488457 and LAAS was over 90%.

### 3.3. Association between PTPN22 Gene Polymorphisms and Microembolic Signals

Ninety-five patients presented with microembolic signals in the LAAS group. There were no differences in the clinical characteristics between the MES-positive and MES-negative groups (Table 3). The outcomes showed that patients carrying the rs2488457 GG genotypes had a higher risk of developing microembolic signals (adjusted OR = 2.591, 95% CI 1.339-5.011, *P* = 0.005). Patients carrying the G allele were more likely to have microembolic signals (adjusted OR = 1.652, 95% CI 1.177-2.319, *P* = 0.004). There was no association between microembolic signals and either rs1217414 or rs2476599 polymorphism (Table 4).

### 3.4. Haplotype Analysis

**Figure 1:**
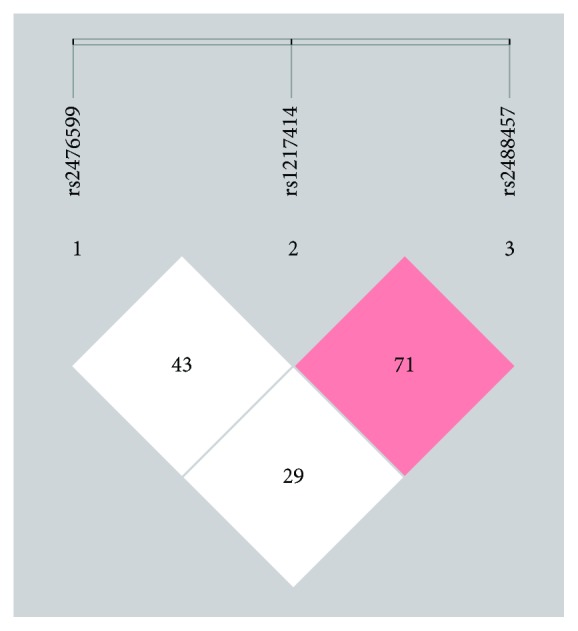
The linkage disequilibrium test of rs2476599, rs1217414, and rs2488457.

Using the linkage disequilibrium (LD) test results, we conducted haplotype analyses for three SNPs using the SHEsis software platform (Figure 1). The results revealed that the prevalence of the GCC haplotype was higher in the control group. In contrast, the frequency of the GTG haplotype was less than the frequency in the LAAS group. The haplotype distributions indicated that the GTG haplotype was related to higher risk of LAAS (OR = 2.778, 95% CI, 1.631–4.731, *P* < 0.001). Nevertheless, the GCC haplotype may play a protective role against LAAS (OR = 0.682, 95% CI, 0.547–0.850, *P* = 0.001) (Table 5).

The *D*′ value between rs1217414 and rs2488457 is 0.71, the *D*′ value between rs2476599 and rs1217414 is 0.43, and the *D*′ value between rs2476599 and rs2488457 is 0.33.

### 3.5. Stratified Analysis

Stratified analyses based on age, gender, and BMI were performed. According to the results, we failed to find any association between these factors and rs2488457 polymorphism (Table 6).

### 3.6. Interaction Analysis

Only the rs2488457 polymorphism was significantly associated with LAAS among the three SNPs. The interaction analysis of the rs2488457 polymorphism with drinking or smoking was performed by means of logistic regression. Compared to nonsmokers with rs2488457 CC genotype, smokers with CG+GG genotype had a distinctly higher risk of LAAS (adjusted OR = 2.492, 95% CI 1.510–4.114, *P* < 0.001). However, there were no any differences in the risk of LAAS among the CG+GG genotype nonsmokers and the CC genotype smokers (adjusted OR = 1.464, 95% CI 0.987–2.172, *P* = 0.058; adjusted OR = 1.778, 95% CI 0.972–3.251, *P* = 0.062, respectively). No interaction was observed between the rs2488457 polymorphism and drinking (Table 7).

## 4. Discussion

To date, our research was the first to explore the relationship between *PTPN22* SNPs (rs2476599, rs1217414, and rs2488457) and LAAS. The results demonstrated that the frequency of G allele in the *PTPN22* rs2488457 was higher in the LAAS group, which suggested that G allele might be a potential risk marker for the prediction of LAAS. However, the present study failed to find any effect of rs2476599 and rs1217414 on LAAS. In the linkage disequilibrium analysis, an incomplete linkage disequilibrium of the three loci was identified. Haplotype analysis revealed that the GTG haplotype might be responsible for the susceptibility to LAAS and that the GCC haplotype might confer a decreased risk of LAAS.

The *PTPN22* gene encodes Lyp, which regulates the production of inflammatory cytokines by participating in the TLR (Toll like receptor) signaling pathway [24–26]. Several SNPs of the *PTPN22* gene, related to a variety of inflammatory diseases, have been found in previous studies [27–29]. In a multicenter cohort study of the Finns, Pertovaara et al. discovered that *PTPN22* polymorphisms were significantly related to increased carotid artery intima-media thickness (IMT) [30]. Saccucci et al. showed that *PTPN22* genetic polymorphisms were related to the occurrence of CAD [31]. In our study, a correlation between the rs2488457 polymorphism and LAAS was found. Nevertheless, the molecular mechanism of how *PTPN22* gene polymorphisms affect atherosclerosis remains unclear. Studies have shown that *PTPN22* increases both the inflammatory response and IL1B (interleukin 1 beta) secretion by dephosphorylating *NLRP3* (NLR family pyrin domain-containing 3) inflammasome to protect *NLRP3* from degradation, which leads to the occurrence of many inflammatory diseases [32, 33]. The *NLRP3* inflammasome and the IL (interleukin) family cytokines are central mediators of vascular inflammation during the process of atherosclerosis [34]. The activation of *NLRP3* inflammasome demands an initial priming signal via receptors that activate NF-*κ*B-mediated transcription, such as TLR or IL [35]. When the *NLRP3* inflammasome is activated, it triggers the excretion of mature, proinflammatory cytokines [36]. Therefore, we speculate that this may be the molecular mechanism of the *PTPN22* genetic variations that influence the occurrence of LAAS.

To study the relationship between *PTPN22* gene polymorphisms and the stability of atherosclerotic plaques, LAAS subjects were split into MES-negative and MES-positive groups according to the presence of MES. Additional analysis showed that patients carrying the G allele were more likely to have MES, thus demonstrating that the rs2488457 polymorphism was involved in the generation of MES. MES indicate that the plaque is unstable. Aubry et al. [37] reported that patients with rheumatoid arthritis (RA) have an increased incidence of vulnerable plaques in the left anterior descending coronary artery, compared with controls, along with a greater evidence of inflammation and plaque instability. Therefore, we hypothesized that the rs2488457 polymorphism results in the instability of atherosclerotic plaques via the acceleration of inflammation.

Smoking and drinking are known to have marked impacts on the progression of atherosclerosis. The proportions of drinking and smoking individuals were larger in the LAAS group, and a strong interaction between smoking and the rs2488457 polymorphism was observed, whereas there was no interaction between the rs2488457 polymorphism and drinking in our study. Nordskog et al. [38] revealed that the expression of proinflammatory mediators was significantly increased in the endothelial cells after exposure to cigarette smoke. An increasing number of scholars believe that vascular inflammation is induced by smoking [39]. Notably, our study revealed that only the combination of smoking status with the rs2488477 G allele (CG+GG) increased the risk of LAAS. This result seemed to imply that individual responses to environmental factors varied by different genetic backgrounds. Genetic and environmental factors are related to each other and can jointly affect the occurrence and development of diseases. However, the potential mechanism of the rs2488457-smoking interaction in LAAS risk is not understood. Future studies focusing on this interaction should be carried out.

## 5. Conclusion

In conclusion, our study indicated that the rs2488457 polymorphism was likely to be associated with LAAS and MES, and this polymorphism had an interaction with smoking in terms of the impacts on LAAS risk. These results could lead to a potential treatment strategy for LAAS, such as gene therapy. However, the current study contains a relatively small sample size, which may be a limitation. Additionally, there were only three SNP loci analyzed, which did not reflect the function of the entire gene. Moreover, the distributions and functions of genes among different ethnicities are different. Our study was only conducted in the Han Chinese population; thus, these conclusions may not represent the whole population. The replication of our study in different populations and the complete analysis of genetic changes in vivo are required to better clarify the effects of *PTPN22* gene polymorphisms on LAAS and MES.

## Figures and Tables

**Table 1:** Clinical characteristics of all the participants.

Variables	LAAS group (*n* = 364)	Control group (*n* = 369)	*P* value
Age (years)	63.33 ± 10.84	62.59 ± 10.13	0.339
Sex (man, %)	271 (74.5%)	265 (71.8%)	0.421
BMI (kg/m^2^)	23.87 ± 3.55	23.65 ± 3.42	0.393
Smoking (*n*, %)	179 (49.2%)	86 (23.3%)	<0.001
Drinking (*n*, %)	154 (42.3%)	85 (23.0%)	<0.001
Family history of IS (*n*, %)	78 (21.4%)	29 (7.9%)	<0.001
Hypertension (*n*, %)	245 (67.3%)	162 (43.9%)	<0.001
Diabetes (*n*, %)	119 (32.7%)	65 (17.6%)	<0.001
CAD (*n*, %)	73 (20.1%)	82 (22.2%)	0.472
Dyslipidemia (*n*, %)	124 (34.1%)	80 (21.7%)	<0.001
GLU (mmol/L)	6.37 ± 1.88	5.58 ± 1.97	<0.001
TG (mmol/L)	1.60 ± 0.70	1.64 ± 0.79	0.469
TC (mmol/L)	4.82 ± 1.11	4.50 ± 1.02	<0.001
HDL (mmol/L)	1.23 ± 0.27	1.25 ± 0.34	0.379
LDL (mmol/L)	2.70 ± 0.70	2.67 ± 0.82	0.595

BMI: body mass index; CAD: coronary artery disease; GLU: serum blood glucose; TG: triglycerides; TC: total cholesterol; HDL: high-density lipoprotein; LDL: low-density lipoprotein.

**Table 2:** Genotype and allelic frequencies of *PTPN22* SNPs in LAAS and control subjects.

SNP site		LAAS group *n* (%)	Control group *n* (%)	Univariate analysis OR (95% CI)^a^	*P* value	Multivariate analysis adjusted OR (95% CI)^b^	*P* value
rs2476599							
Genotype	GG	323 (88.7%)	331 (89.7%)	1.000 (reference)	-	1.000 (reference)	-
	GA	38 (10.4%)	37 (10.0%)	1.052 (0.653-1.697)	0.834	0.993 (0.591-1.669)	0.978
	AA	3 (0.8%)	1 (0.3%)	3.074 (0.318-29.709)	0.603	5.057 (0.435-58.812)	0.195
	GA+AA	41 (11.3%)	38 (10.3%)	1.106 (0.693-1.764)	0.673	1.069 (0.643-1.778)	0.797
Allele	G	684 (94.0%)	699 (94.7%)	1.000 (reference)	-	1.000 (reference)	-
	A	44 (6.0%)	39 (5.3%)	1.153 (0.740-1.797)	0.529	1.145 (0.706-1.855)	0.584

rs1217414							
Genotype	CC	302 (83.0%)	318 (86.2%)	1.000 (reference)	-	1.000 (reference)	-
	CT	57 (15.7%)	47 (12.7%)	1.277 (0.842-1.938)	0.250	1.233 (0.786-1.935)	0.362
	TT	5 (1.4%)	4 (1.1%)	1.316 (0.350-4.948)	0.943	1.452 (0.340-6.199)	0.614
	CT+TT	62 (17.0%)	51 (13.8%)	1.280 (0.856-1.915)	0.229	1.249 (0.808-1.931)	0.317
Allele	C	661 (90.8%)	683 (92.5%)	1.000 (reference)	-	1.000 (reference)	-
	T	67 (9.2%)	55 (7.5%)	1.259 (0.867-1.827)	0.225	1.242 (0.830-1.859)	0.292

rs2488457							
Genotype	CC	125 (34.3%)	168 (45.5%)	1.000 (reference)	-	1.000 (reference)	-
	CG	167 (45.9%)	161 (43.6%)	1.394 (1.015-1.914)	0.040	1.244 (0.881-1.757)	0.215
	GG	72 (19.8%)	40 (10.8%)	2.419 (1.542.-3.796)	<0.001	2.275 (1.395-3.709)	0.001
	CG+GG	239 (65.7%)	201 (54.5%)	1.598 (1.186-2.153)	0.002	1.444 (1.044-1.997)	0.027
Allele	C	417 (57.3%)	497 (67.3%)	1.000 (reference)	-	1.000 (reference)	-
	G	311 (42.7%)	241 (32.7%)	1.538 (1.243-1.903)	<0.001	1.456 (1.156-1.833)	0.001

^a^Univariate analysis; ^b^multivariate analysis (covariates: sex, age, history of smoking and drinking, hypertension, diabetes, dyslipidemia, and family history of ischemic stroke events).

**Table 3:** Clinical characteristics of MES (+) and MES (−) groups.

Variables	MES (+) (*n* = 95)	MES (-) (*n* = 269)	*P* value
Age (years)	63.42 ± 10.97	63.30 ± 10.81	0.926
Sex (man, %)	70 (73.7%)	201 (74.7%)	0.842
BMI (kg/m^2^)	24.23 ± 3.56	23.74 ± 3.55	0.249
Smoking (*n*, %)	39 (41.1%)	140 (52.0%)	0.065
Drinking (*n*, %)	36 (37.9%)	118 (43.9%)	0.311
Family history of IS (*n*, %)	22 (23.2%)	56 (20.8%)	0.633
Hypertension (*n*, %)	59 (62.1%)	186 (69.1%)	0.209
Diabetes (*n*, %)	30 (31.6%)	89 (33.1%)	0.788
CAD (*n*, %)	21 (22.1%)	52 (19.3%)	0.562
Dyslipidemia (*n*, %)	30 (31.6%)	94 (34.9%)	0.552
GLU (mmol/L)	6.43 ± 1.83	6.35 ± 1.90	0.729
TG (mmol/L)	1.63 ± 0.77	1.59 ± 0.68	0.635
TC (mmol/L)	4.75 ± 1.15	4.84 ± 1.09	0.482
HDL (mmol/L)	1.22 ± 0.26	1.23 ± 0.28	0.761
LDL (mmol/L)	2.73 ± 0.66	2.69 ± 0.71	0.631

BMI: body mass index; CAD: coronary artery disease; GLU: serum blood glucose; TG: triglycerides; TC: total cholesterol; HDL: high-density lipoprotein; LDL: low-density lipoprotein.

**Table 4:** Genotypes and allelic frequencies of *PTPN22* SNPs in MES (+) and MES (−) groups.

SNP site		MES (+) *n* (%)	MES (-) *n* (%)	Univariate analysis OR (95% CI)^a^	*P* value	Multivariate analysis adjusted OR (95% CI)^b^	*P* value
rs2476599							
Genotype	GG	84 (88.4%)	239 (88.8%)	1.000 (reference)	-	1.000 (reference)	-
	GA	10 (10.5%)	28 (10.4%)	1.016 (0.473-2.181)	0.967	0.914 (0.418-2.000)	0.823
	AA	1 (1.1%)	2 (0.7%)	1.423 (0.127-15.892)	1.000	1.249 (0.109-14.291)	0.858
	GA+AA	11 (11.6%)	30 (11.2%)	1.043 (0.501-2.174)	0.910	0.938 (0.442-1.990)	0.867
Allele	G	178 (93.7%)	506 (94.1%)	1.000 (reference)	-	1.000 (reference)	-
	A	12 (6.3%)	32 (5.9%)	1.066 (0.537-2.115)	0.855	0.963 (0.480-1.934)	0.916

rs1217414							
Genotype	CC	77 (81.1%)	225 (83.6%)	1.000 (reference)	-	1.000 (reference)	-
	CT	17 (17.9%)	40 (14.9%)	1.242 (0.666-2.317)	0.495	1.161 (0.613-2.200)	0.647
	TT	1 (1.1%)	4 (1.5%)	0.731 (0.080-6.636)	1.000	0.726 (0.078-6.715)	0.778
	CT+TT	18 (18.9%)	44 (16.4%)	1.195 (0.652-2.192)	0.564	1.123 (0.603-2.093)	0.715
Allele	C	171 (90.0%)	490 (91.1%)	1.000 (reference)	-	1.000 (reference)	-
	T	19 (10.0%)	48 (8.9%)	1.134 (0.649-1.984)	0.659	1.074 (0.608-1.898)	0.806

rs2488457							
Genotype	CC	25 (26.3%)	100 (37.2%)	1.000 (reference)	-	1.000 (reference)	-
	CG	42 (44.2%)	125 (46.5%)	1.344 (0.767-2.354)	0.300	1.465 (0.822-2.611)	0.195
	GG	28 (29.5%)	44 (16.4%)	2.545 (1.335-4.854)	0.004	2.591 (1.339-5.011)	0.005
	CG+GG	70 (73.7%)	169 (62.8%)	1.657 (0.986-2.785)	0.055	1.780 (1.042-3.039)	0.035
Allele	C	92 (48.4%)	325 (60.4%)	1.000 (reference)	-	1.000 (reference)	-
	G	98 (51.6%)	213 (39.6%)	1.625 (1.165-2.267)	0.004	1.652 (1.177-2.319)	0.004

^a^Univariate analysis; ^b^multivariate analysis (covariates: sex, age, history of smoking and drinking, hypertension, diabetes, dyslipidemia, and family history of ischemic stroke events).

**Table 5:** Haplotype analysis of rs2476599, rs1217414, and rs2488457 in LAAS patients and control subjects.

Haplotype	LAAS group (freq)	Control group (freq)	OR	95% CI	*P* value
A C C	15.61 (0.021)	7.85 (0.011)	-	-	-
A C G	13.79 (0.019)	6.69 (0.009)	-	-	-
A T C	0.00 (0.000)	13.72 (0.019)	-	-	-
A T G	14.60 (0.020)	10.75 (0.015)	-	-	-
G C C	400.25 (0.550)	464.43 (0.629)	0.682	0.547-0.850	0.001
G C G	231.35 (0.318)	204.04 (0.276)	1.215	0.968-1.526	0.093
G T C	1.14 (0.002)	11.00 (0.015)	-	-	-
G T G	51.26 (0.070)	19.53 (0.026)	2.778	1.631-4.731	<0.001

Frequencies < 0.03 were ignored in the analysis.

**Table 6:** The risk of rs2488457 for LAAS according to age, gender, and BMI.

Factors	Genotype	Multivariate analysis Adjusted OR (95% CI)	*P* value
Age			
<65	CC	1.000 (reference)	-
	CG+GG	1.347 (0.874-2.076)	0.177
≥65	CC	1.000 (reference)	-
	CG+GG	1.454 (0.688-2.011)	0.169
Gender			
Male	CC	1.000 (reference)	-
	CG+GG	1.207 (0.824-1.734)	0.101
Female	CC	1.000 (reference)	-
	CG+GG	1.311 (0.490-3.511)	0.590
BMI			
<24 kg/m^2^	CC	1.000 (reference)	-
	CG+GG	1.533 (0.993-2.368)	0.054
≥24 kg/m^2^	CC	1.000 (reference)	-
	CG+GG	1.273 (0.551-2.941)	0.571

BMI: body mass index; multivariate analysis (covariates: sex, age, history of smoking and drinking, hypertension, diabetes, dyslipidemia, and family history of ischemic stroke events).

**Table 7:** Interaction analysis of rs2488457 with drinking or smoking by using logistic regression.

SNP site	Smoking			Drinking		
rs2488457		Adjusted OR (95% CI)^a^	*P* value		Adjusted OR (95% CI)^b^	*P* value
CC	No	1.000 (reference)	-	No	1.000 (reference)	-
CC	Yes	1.778 (0.972-3.251)	0.062	Yes	1.278 (0.600-2.721)	0.524
CG+GG	No	1.464 (0.987-2.172)	0.058	No	1.389 (0.944-2.045)	0.096
CG+GG	Yes	2.492 (1.510-4.114)	<0.001	Yes	2.019 (0.997-4.090)	0.051

^a^Adjusted for sex, age, history of drinking, diabetes, hypertension, dyslipidemia, and family history of ischemic stroke events; ^b^adjusted for sex, age, history of smoking, hypertension, diabetes, dyslipidemia, and family history of ischemic stroke events.

## Data Availability

The data used to support the findings of this study are available from the corresponding authors upon request.

## Supplementary Materials

Supplementary 1 Table S1: the sequences of the primers and probes for TaqMan technology.

Supplementary 2 Table S2: the sequences of the primers for DNA sequencing.
