# Supplementary material for: PTPN22 Gene Polymorphisms Are Associated with Susceptibility to Large Artery Atherosclerotic Stroke and Microembolic Signals
Source: Dis Markers. 2019 May 5;2019:2193835. doi: 10.1155/2019/2193835 (PMC6525845; doi:10.1155/2019/2193835)
Supplement: Supplementary 1 — Table S1: the sequences of the primers and probes for TaqMan technology. [file 2193835.f1.doc]

**Table S1:** The sequences of the primers and probes for TaqMan technology.

| SNP site | Primer and probe | Sequence 5’ to 3’ |
| --- | --- | --- |
| rs2476599 | Forward | TGTGAACTGCATATGCATATTAGG |
|  | Reverse | CTCCTCGAAAAGGAAGGTGTT |
|  | FAM-labeled probe | ACATGCATACATAATAC |
|  | VIC-labeled probe | ACATGCATACGTAATAC |
| rs1217414 | Forward | ACAGCGGTGAACAAAAAGAAC |
|  | Reverse | GTGTTTTCACATCTGCTATCCG |
|  | FAM-labeled probe | TACCAGAGTGTTAGCTC |
|  | VIC-labeled probe | CCAGAATGTTAGCTCC |
| rs2488457 | Forward | TGAATTAGGCACTTGGGTAGACT |
|  | Reverse | AGGCAGGCTCTTCTAGAAAAAC |
|  | FAM-labeled probe | CACATTTGAGCTTGCAT |
|  | VIC-labeled probe | CACATTTCAGCTTGCAT |
|  |  |  |
|  |  |  |
