# Supplementary material for: PTPN22 Gene Polymorphisms Are Associated with Susceptibility to Large Artery Atherosclerotic Stroke and Microembolic Signals
Source: Dis Markers. 2019 May 5;2019:2193835. doi: 10.1155/2019/2193835 (PMC6525845; doi:10.1155/2019/2193835)
Supplement: Supplementary 2 — Table S2: the sequences of the primers for DNA sequencing. [file 2193835.f2.doc]

**Table S2:** The sequences of the primers for DNA sequencing.

| SNP site | Primer and probe | Sequence 5’ to 3’ | Fragment size |
| --- | --- | --- | --- |
| rs2476599 | Forward | TGGCAATGGAAAAAGATAATTTCAGGT | 398bp |
|  | Reverse | GCCTACAGCAATGCTTAGCACA |  |
| rs1217414 | Forward | ACATGTCCTACCTATCCTCACAGG | 357bp |
|  | Reverse | ACCAGAGGGGCTCAGACTGA |  |
| rs2488457 | Forward | ACATTTGTTGAACTTCTGCCTTCAA | 376bp |
|  | Reverse | GGAGCACCTGACCAGACAGT |  |
|  |  |  |  |
|  |  |  |  |
